# Homeostatic Appetite and Hedonic Hunger 13 Years After Roux-en-Y Gastric Bypass: Potential Associations and Predictive Value in Determining Long-Term Weight Loss Outcomes

**DOI:** 10.1007/s11695-025-07955-w

**Published:** 2025-06-09

**Authors:** Siren Nymo, Julianne Lundanes, Jens Frederik Rehfeld, Jens Juul Holst, Sten Madsbad, Carsten Dirksen, Kirstine Nyvold Bojsen-Møller, Jorunn Sandvik, Catia Martins

**Affiliations:** 1 Department of Public Health and Nursing, Faculty of Medicine and Health Sciences, Norwegian University of Science and Technology (NTNU), Trondheim, Norway; 2 Centre for Obesity and Innovation, Clinic of Surgery, St. Olavs University Hospital, Trondheim, Norway; 3 Nord-Trøndelag Hospital Trust, Clinic of Surgery, Namsos Hospital, Namsos, Norway; 4 Obesity Research Group, Department of Clinical and Molecular Medicine, Faculty of Medicine and Health Sciences, Norwegian University of Science and Technology (NTNU), Trondheim, Norway; 5 Department of Clinical Biochemistry, Rigshospitalet, University of Copenhagen, Copenhagen, Denmark; 6 NNF Center for Basic Metabolic Research and Department of Biomedical Sciences, The Panum Institute, University of Copenhagen, Copenhagen, Denmark; 7 Department of Respiratory Diseases and Endocrinology, Copenhagen University Hospital – Amager and Hvidovre AND Department of Clinical Medicine, University of Copenhagen, Copenhagen, Denmark; 8 Møre and Romsdal Hospital Trust, Clinic of Surgery, Ålesund Hospital, Trondheim, Norway; 9 Department of Nutrition Sciences, University of Alabama at Birmingham (UAB), Birmingham, AL USA

**Keywords:** Homeostatic appetite, Hedonic hunger, Power of food scale, Prospective food consumption

## Abstract

**Introduction:**

The interplay between homeostatic and hedonic appetite following Roux-en-Y gastric bypass (RYGB) and their potential relevance in modulating long-term weight loss (WL) outcomes has not been properly explored.

**Aim:**

The main aim of this analysis was to explore the association between homeostatic appetite markers and hedonic hunger 13 years post-RYGB. A secondary aim was to determine the association between homeostatic and hedonic appetite, and % total weight loss (TWL).

**Methods:**

Hedonic hunger was measured with the Power of Food scale (food available, food present, food tasted and aggregated score). The plasma concentration of gastrointestinal (GI) hormones involved in appetite regulation was measured with validated methods, and subjective appetite ratings (hunger, fullness, desire to eat (DTE), and prospective food consumption (PFC)), with visual analogue scales, both in the fasting and postprandial states.

**Results:**

Forty-five participants (age: 50.7 ± 7.8 years, BMI: 34.8 ± 9.3 kg/m^2^, %TWL: 21.0 ± 17.0) were included. Postprandial GLP-1 was inversely associated with food available score. DTE was positively correlated with food available, while PFC was positively correlated with food available, food present, and aggregated score. After adjusting for covariates, food available together with PFC ratings explained 30% of the variability in %TWL post-RYGB. Hormones were found not to contribute to %TWL.

**Conclusion:**

The present analyses suggest that the hedonic and homeostatic appetite control systems are intertwined and are both important in modulating long-term WL outcomes post-RYGB. The measurement of appetite ratings and hedonic hunger might be clinically relevant, both during screening and post-operative follow-up aiming at improving long-term WL outcomes.

**Supplementary Information:**

The online version contains supplementary material available at 10.1007/s11695-025-07955-w.

## Introduction

Successful long-term weight loss (WL) maintenance remains the biggest challenge in obesity management [1, 2], and that also holds true for bariatric surgery [3–5]. Despite the success of Roux-en-Y gastric bypass (RYGB) in inducing clinically relevant WL, approximately 30% of the patients experience suboptimal WL (< 20% of total weight loss (TWL)) in the long term, and the exact reasons for this phenomenon remain to be fully elucidated [6].

Appetite control involves a complex interplay between internal and external sensory information, emotions, and cognitions, with the ultimate goal of meeting biological needs [7]. Homeostatic appetite regulation involves both tonic signals that circulate in direct proportion to the amount of fat stores (leptin) and tonic signals (ghrelin, glucagon-like peptide 1 (GLP-1), peptide YY (PYY), and cholecystokinin (CCK)) that oscillate with each meal. The overall aim of the homeostatic system is to maintain energy balance (EB). Subjective ratings of appetite, including hunger, fullness, desire to eat (DTE), and prospective food consumption (PFC) are valid and reliable measures of the strength of the motivation to eat [8]. The hedonic appetite control system, on the other hand, is mainly reward-driven and can operate independently of homeostatic needs [9]. When food is highly palatable and easily available, people tend to overeat [10].

Some studies suggest that suboptimal WL following RYGB is the result of a weaker satiety response to a meal, namely a blunted GLP-1 postprandial secretion [11, 12]. Our research group has previously shown that suboptimal WL following RYGB is associated with lower basal and postprandial ghrelin plasma concentrations, a weaker postprandial GLP-1 response, as well as higher hunger ratings in the fasting state, and higher postprandial DTE and PFC ratings [13], and that these altered responses were associated with long-term WL outcomes [13]. However, a recent study showed no difference in the postprandial concentrations of GLP-1, PYY, or CCK between those experiencing optimal and suboptimal WL post-RYGB, and suggested an impaired central anorectic response to gut hormones as the culprit for WL failure [14].

Regarding a possible role for hedonic appetite in modulating long-term WL post-bariatric surgery, the evidence seems to be more consistent. A reduction in hedonic hunger has been reported following bariatric surgery [15], and our group recently showed that suboptimal WL post-RYGB was associated with higher hedonic hunger [16]. Additionally, low levels of disinhibition and hunger after surgery have been shown to predict better WL outcomes 10 years after RYGB [17]. Even though the evidence is inconclusive, most of the studies show that RYGB promotes a reduction in the drive to consume palatable foods and leads to an increased desire for lower-energy dense foods and improved dietary habits [15, 18–22].

However, a potential interplay between homeostatic and hedonic appetite in modulating long-term WL outcomes post-RYGB has not been explored. Therefore, this analysis aims to explore the association between homeostatic appetite markers and hedonic hunger in patients who underwent RYGB more than 10 years ago. A secondary aim was to determine the potential association between homeostatic and hedonic appetite and %TWL.

## Methods

### Study Design and Participants

The present manuscript represents an exploratory analysis using data from a cross-sectional case–control study named the Bariatric Surgery Observation Study (BAROBS), on participants who underwent RYGB between 2003 and 2009 with a follow-up in 2020 and 2021 [13]. The primary objective of the BAROBS study was to explore long-term effects after RYGB.

#### Assessments

Anthropometric measurements were taken in the fasting state (12 h), and appetite markers were assessed before and after a standardized liquid breakfast meal (Diben shake (200 ml: 300 kcal, 15 g protein, 14 g fat, and 26 g carbohydrates) (Fresenius Kabi, Bad Homburg, Germany)).

### Body Weight and Composition

Body weight and composition were assessed with air-displacement plethysmography (BodPod, Cosmed, Concord, CA, USA), using the Brozeq equation [23]. Initial weight was the participants’ weight closest to the time of surgery. Total WL was calculated from the weight closest to the operation until the follow-up. %TWL was estimated using standard equations [24].

### Appetite Markers

#### Hedonic Hunger

Hedonic hunger was measured with the Power of Food scale (PFS) in the fed state (immediately after breakfast). This questionnaire measures hedonic hunger by assessing individual differences in appetitive responsiveness to highly palatable foods [25]. It is divided into three categories: food available, which measures thoughts about food in general; food present, which measures attraction to food when food is directly available; and food tasted, which assesses the desire for food when first tasted [26]. A mean score was calculated for each category, in addition to an aggregated domain, which is the mean score for all three categories [27]. A higher score indicates a higher hedonic hunger.

#### Homeostatic Appetite Markers: Appetite Hormone and Subjective Appetite Ratings

Blood samples for the quantification of the plasma concentrations of gastrointestinal (GI) hormones, as well as subjective appetite ratings, with visual analogue scales, were collected in fasting, and every 30 min for 2.5 h after a standardized liquid breakfast (previously described). The methods of analyses of the hormones (active ghrelin, total PYY, total GLP-1, and CCK) and subjective ratings of hunger, fullness, DTE, and PFC have been previously described [13, 28].

### Statistical analysis

Statistical analysis was performed using IBM SPSS Statistics 29 (SPSS In., Chicago, IL, USA), and data presented as mean ± SD, unless otherwise stated. All variables were checked for normality with Shapiro–Wilk test and visual inspection of histograms and Q-Q plots. Statistical significance was set at *p* < 0.05.

The trapezoidal rule was used to calculate total area under the curve (tAUC) from 0 to 150 min for all appetite hormones and subjective appetite feelings. The association between homeostatic and hedonic appetite markers was performed with Pearson or Spearman correlation, depending on the normality of the data. In addition, multiple linear regression was used to determine if homeostatic and hedonic appetite variables were significant predictors of long-term WL outcomes (TWL) after adjusting for age, sex, and pre-operative BMI. *β*-coefficients are reported as unstandardized estimates and 95% CI.

## Results

### Participants

Data from 45 participants (91% females) was included in this secondary analysis. Participants’ characteristics are presented in Table 1. Participants had a mean age of 51 ± 8 years, a BMI of 35 ± 9 kg/m^2^, and a %TWL of 21 ± 17% (min −17 and max 55%).

**Table 1:** General characteristics of the participants

Female, *n* (%)	45 (91)
Age, years	50.8 ± 7.8
BMI, kg/m^2^	34.9 ± 9.3
Weight, kg	99.2 ± 29.6
Preoperative BMI, kg/m^2^	44.3 ± 5.1
%TWL	20.5 ± 16.9

Data presented as mean ± SD

*BMI* body mass index, *TWL* total weight loss

### Association Between Homeostatic and Hedonic Appetite Markers

Correlation analyses between hedonic hunger, plasma concentrations of appetite hormone and subjective appetite rating are presented in Supplementary Table 1 and 2, respectively. Postprandial GLP-1 concentrations were inversely associated with food available (iAUC: *r* = − 0.351, *p* = 0.018, *n* = 45) and aggregated score (iAUC: *r* = − 0.301, *p* = 0.045, *n* = 45), while basal CCK concentration was positively associated with food available (0.314, *p* = 0.038, *n* = 44) (see Fig, 1 A–C).

**Fig. 1:**
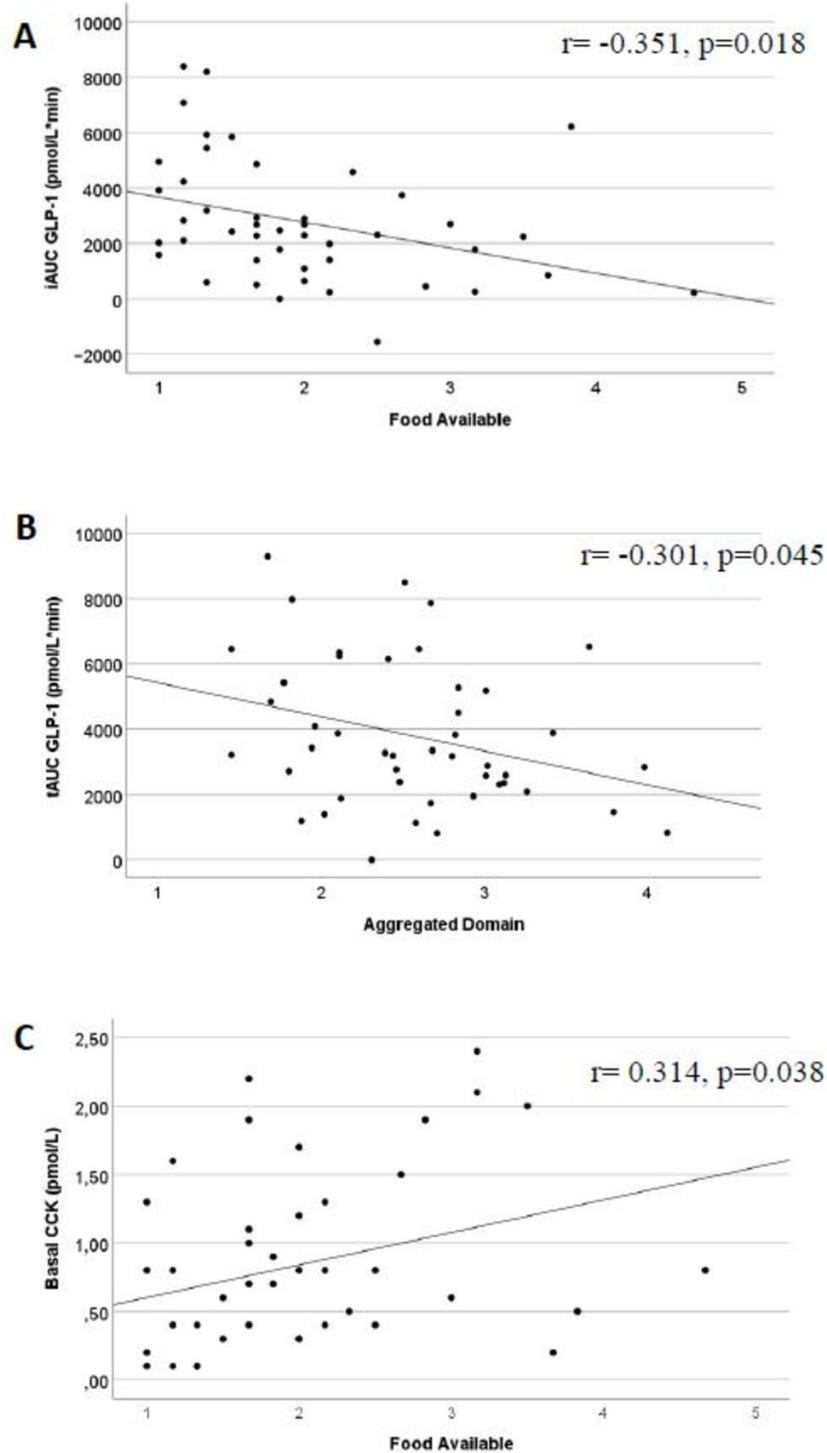
Scatterplots for the association between hedonic hunger and postprandial concentrations of GLP-1 (**A** and **B**) and basal concentrations of CCK (**C**) 13 years after RYGB. CCK, cholecystokinin; GLP-1, glucagon-like peptide-1; iAUC, total area under the curve; RYGB, Roux-en-Y gastric bypass

**Fig. 2:**
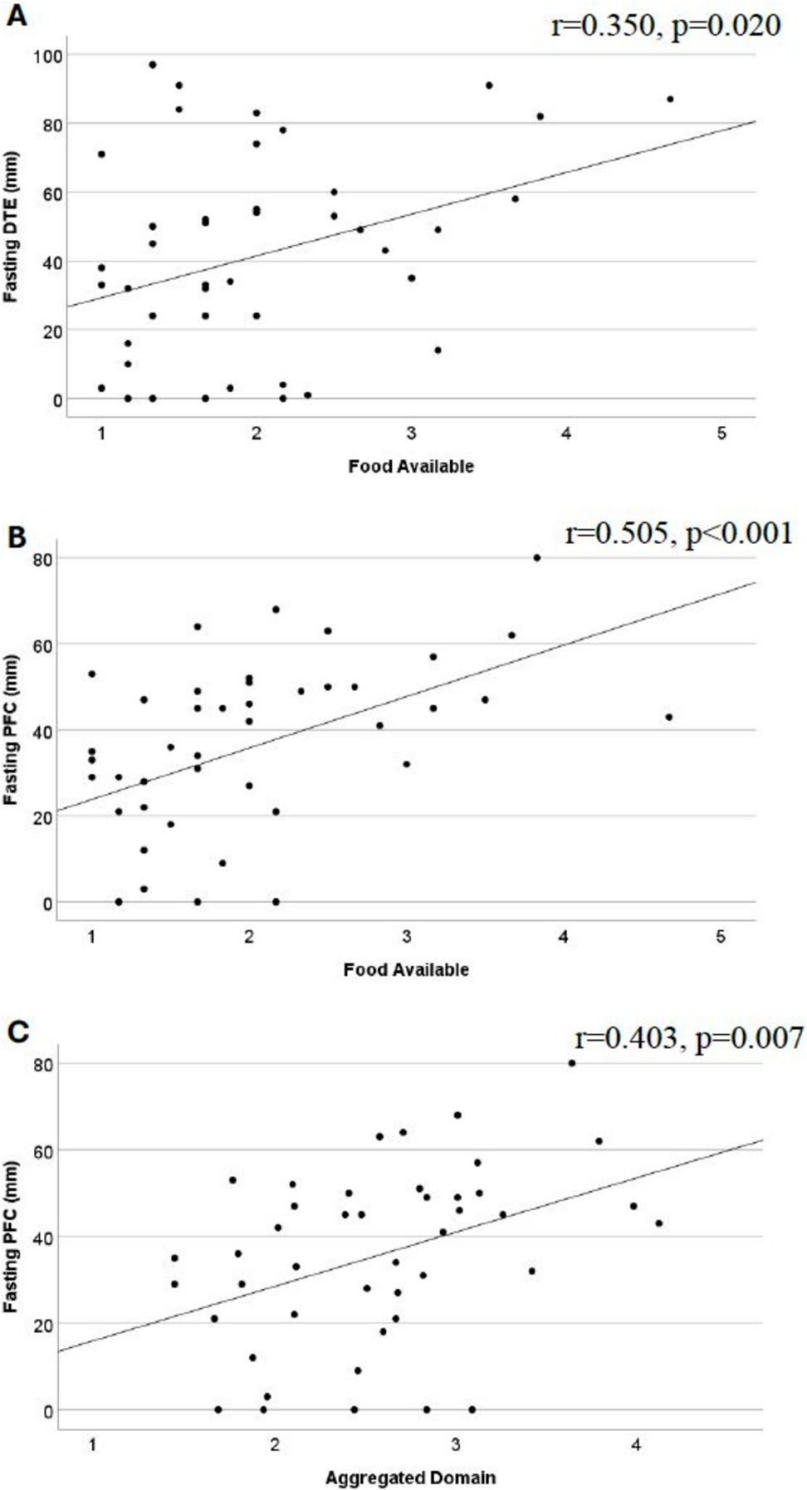
Scatterplots for the association between hedonic hunger and subjective ratings of desire to eat (**A**) and prospective food consumption (**B** and **C**) in the fasting state more than 13 years after RYGB. DTE, desire to eat; PFC, prospective food consumption; RYGB, Roux-en-Y gastric bypass

Ratings of DTE and PFC in the fasting state were positively correlated with food available (*r* = 0.350, *p* = 0.020, *n* = 44 and *r* = 0.505, *p* < 0.001, *n* = 44, respectively). Ratings of PFC in the fasting state were positively correlated with aggregated score (*r* = 0.403, *p* = 0.007, *n* = 44) (see Fig. 2 A–C).

### Regression Analyses

Multivariate linear regression models predicting long-term %TWL from homeostatic and hedonic appetite variables are presented in Table 2 and Supplementary Table 3. After adjusting for age, sex, and preoperative BMI, food available explained 23%, ratings of PFC in the fasting state explained 28%, and food available together with ratings of PFC in the fasting state explained 30% of the variability in %TWL 13 years post-RYGB. The plasma concentration of hormones was not found to contribute significantly to %TWL.

**Table 2:** Multivariate linear regression models predicting TWL after RYGB

Models	*ß*-coefficient (95% CI)	*P*-value	Adjusted *R*^2^
Model A		0.006	0.227
Constant	43.712 (− 13.626, 101.051)	0.131	
Age	0.459 (− 0.136, 1.055)	0.127	
Sex	− 9.481 (− 25.172, 6.209)	0.229	
Preoperative BMI	− 0.519 (− 1.410, 0.373)	0.247	
**PFS-FA**	− 7.693 (− 13.136, − 2.250)	*0.007*	
Model B		0.002	0.300
Constant	41.116 (− 12.007, 94.239)	0.125	
**Age**	0.608 (0.048, 1.169)	*0.034*	
Sex	− 10.271 (− 24.793, 4.252)	0.160	
Preop BMI	− 0.624 (− 1.455, 0.208)	0.208	
**PFS-FA**	− 5.678 (− 11.562, 0.206)	0.058	
PFC-fasting	− 0.125 (− 0.367, 0.117)	0.302	
Model C		0.002	0.276
Constant	28.691 (− 21.686, 79.068)	0.257	
**Age**	0.762 (0.212, 1.312)	*0.008*	
Sex	− 9.599 (− 24.490, 5.292)	0.200	
Preop BMI	− 0.660 (− 1.496, 0.175)	0.118	
**PFC-fasting**	− 0.269 (− 0.475, − 0.063)	*0.012*	

*TWL* total weight loss, *RYGB* Roux-en-Y gastric bypass, *PFC* prospective food consumption, *PFS-FA* food available

Variance inflation factors (VIF) < 1.8

## Discussion

The aim of the present analyses was to explore the interplay between homeostatic and hedonic appetite in modulating long-term WL after RYGB. Patients with a smaller postprandial GLP-1 response, as well as those with higher ratings of DTE or PFC in the fasting state, reported more thoughts about food in general (higher food available scores from PFS). Furthermore, “thoughts about food in general” explained 23% of the variability in %TWL 13 years post-RYGB, ratings of PFC in fasting 28%, and both variables together up to 30%.

Hedonic hunger, both aggregated scores, as well as the subdomains food available and food tasted, has been reported to decrease 16 months post-RYGB, and absolute WL to be inversely associated with food tasted [18]. Most patients reach nadir weight at this time point and experience several metabolic improvements [5]. Although the direction of causality is difficult to assess, the fact that hedonic hunger can be modified by WL indicates that high hedonic hunger might be a consequence of obesity [29]. The ability to manage food urges is thought to be an important part of successful WL treatment, and unfortunately, changes in postoperative appetite and eating urges following RYGB vary greatly among patients [30]. The very large variability in WL outcomes (mean %TWL: 21 ± 17, min − 17 and max 55%) found in the present study might have contributed to the variability in hedonic hunger scores.

In the present analysis, those with a blunted postprandial GLP-1 response reported increased preoccupation with food. Variability in GLP-1 response has been documented in a previous cross-sectional study from our group, and a smaller postprandial GLP-1 response linked to suboptimal WL [13]. The simultaneous occurrence of reduced GLP-1 response and heightened food preoccupation may contribute to disturbed eating patterns, potentially predisposing individuals to overconsumption and subsequent weight regain or suboptimal WL [13]. These findings are supported by a study that looked at the interaction between GLP-1 and dorsolateral prefrontal cortex (DLPFC) activity with food-cue reactivity. The authors found that neither GLP-1 nor the DLPFC responses individually predicted WL; however, their interaction was a robust predictor of WL over time [31]. A lower activation of several brain reward systems, namely nucleus accumbens, in response to food, especially high-energy dense foods, has also been found after RYGB [32]. These changes in the brain reward system may translate into beneficial food choices and reduced hedonic hunger post-bariatric surgery. This is in line with the higher hedonic hunger seen in individuals with suboptimal WL after RYGB [16] and indicates a more dysfunctional eating behaviour in this subgroup of bariatric patients.

In the present study, individuals with higher ratings of DTE and PFC in the fasting state also reported more thoughts about food in general. We have previously shown that suboptimal WL following RYGB is characterized by greater ratings of PFC and DTE [13], as well as a higher intake of palatable energy-dense foods [22]. In general, RYGB is associated with decreased DTE of highly palatable food [33, 34] and participants with greater WL report a stronger desire (and consumption) for high-protein food [35].

Individual differences in hedonic hunger can be attributed to various factors such as metabolic, physiological, psychological, and genetic influences, all of which are part of the integrated psychobiological appetite system [36]. The interplay between the homeostatic and hedonic appetite regulation represents two parts of the same interrelated brain system. However, hedonic hunger does not always translate into increased food intake [26], as individuals experiencing higher hedonic hunger are often thinking about eating, without necessarily consuming food [26].

In the presented study, there was no correlation between hunger ratings in the fasting state and hedonic hunger, and hunger ratings were not a significant predictor of %TWL (data not shown). This supports the idea that the hedonic appetite control system can work independently of biological hunger and may lead to overeating [10]. Hedonic hunger has been associated with greater eating in the absence of hunger [37]. In the presented study, food available (thoughts about food in general) explained 23% and subjective ratings of PFC in the fasting state 28% of the variability in %TWL post-RYGB. Interestingly, when both variables were put together in the same model, up to 30% of the variability in %TWL could be explained.

WL may lead to an improvement in appetite regulation, as seen by a normalization in several homeostatic appetite markers following WL [38]. Therefore, it may be plausible that a normalization of hedonic hunger might also occur with WL. In a recent observational study, hedonic hunger was reduced following an average 16% WL, independently of how WL was achieved (diet, RYGB, or sleeve gastrectomy) [39]. Even though this was a short-term study (11 weeks), another study comparing different bariatric procedures (1 year) found a reduced drive to consume for pleasure in the absence of physical hunger after both RYGB and sleeve gastrectomy [40]. Increased hedonic hunger was also found in individuals with severe obesity compared to non-obese controls, but this was not seen after bariatric surgery [15], again suggesting a normalization of the reward appetite control system with WL.

This study presents several strengths. First, the follow-up period post-RYGB was very long (13 years). Second, different aspects of the complex appetite control system were measured, including hedonic hunger, fasting and postprandial concentrations of several appetite hormones, and subjective appetite ratings. However, this study also presents some limitations. First, it represents a secondary analysis and, therefore, was not powered to investigate the interplay between homeostatic appetite and hedonic hunger. As such, these analyses are exploratory in nature and need to be confirmed in future studies. Second, the test meal used was a liquid oral supplement, not a mixed meal, and cannot be defined as a palatable meal. Despite this, several domains of hedonic hunger were associated with GLP-1 postprandial secretion and subjective ratings of appetite, and hedonic hunger was found to be a significant predictor of %TWL.

The findings of the present analyses suggest that the assessment of subjective appetite ratings and hedonic hunger might be clinically relevant, both during screening for bariatric surgery, as well as during post-operative follow-up, aiming at improving long-term WL outcomes. Patients with a phenotype characterized by a weaker satiety response to a meal and more preoccupation with food are likely more vulnerable and need more attention in clinical practice to maximize WL and avoid weight regain in the long term. Both subjective appetite ratings and hedonic hunger are easy to measure in clinical settings and could be helpful tools for personalizing obesity treatment, by adding behavior programs and/or incretin-mimetic drugs, namely GLP-1 receptor agonists, or drugs that target the brain’s reward pathways in those patients at risk for suboptimal WL post-RYGB.

## Conclusion

The present analyses suggest that the hedonic and homeostatic appetite control systems are intertwined and are both important in modulating long-term WL outcomes post-RYGB. The desire to consume available foods and subjective ratings of PFC seem to be especially important in explaining variability in long-term WL outcomes. These appetite profiles can be easily measured in clinical practice and be part of the toolkit to help guide personalized treatment and improve long-term WL outcomes post-RYGB.

## Supplementary Information

Below is the link to the electronic supplementary material.

Supplementary file 1 (DOCX 32.9 KB)

Supplementary file 2 (DOCX 33.0 KB)

Supplementary file 3 (DOCX 22.1 KB)

## Data Availability

Data is provided within the manuscript and supplementary files. Further data is available upon request.
