# Supplementary material for: Homeostatic Appetite and Hedonic Hunger 13 Years After Roux-en-Y Gastric Bypass: Potential Associations and Predictive Value in Determining Long-Term Weight Loss Outcomes
Source: Obes Surg. 2025 Jun 9;35(7):2719–26. doi: 10.1007/s11695-025-07955-w (PMC12270954; doi:10.1007/s11695-025-07955-w)
Supplement: Supplementary file 1 — (DOCX 32.9 KB) [file 11695_2025_7955_MOESM1_ESM.docx]

| Supplementary table 1. Correlations between the concentrations of appetite related hormones and hedonic hunger after RYGB | | | | | | | | | | | | |
| --- | --- | --- | --- | --- | --- | --- | --- | --- | --- | --- | --- | --- |
|  | PFS – FA | | | PFS-FP | | | PFS-FT | | | PFS-AS | | |
|  | r | P | n | r | P | n | r | P | n | r | P | n |
| Basal AG, pmol/L | -0.231 | 0.132 | 44 | -0.190 | 0.217 | 44 | -0.073 | 0.639 | 44 | -0.212 | 0.166 | 44 |
| AG iAUC, pmol/L*min | 0.268 | 0.075 | 45 | 0.184 | 0.226 | 45 | -0.018 | 0.907 | 45 | 0.195 | 0.199 | 45 |
| Basal GLP-1, pmol/L | -0.039 | 0.804 | 44 | -0.059 | 0.702 | 44 | 0.065 | 0.677 | 44 | -0.023 | 0.882 | 44 |
| GLP-1 iAUC, pmol/L*min | -0.351 | **0.018** | 45 | -0.291 | 0.052 | 45 | -0.037 | 0.810 | 45 | -0.301 | **0.045** | 45 |
| Basal PYY, pmol/L | -0.118 | 0.444 | 44 | -0.034 | 0.827 | 44 | 0.092 | 0.553 | 44 | -0.036 | 0.819 | 44 |
| PYY iAUC, pmol/L*min | -0.117 | 0.443 | 45 | -0.101 | 0.508 | 45 | -0.019 | 0.899 | 45 | -0.105 | 0.491 | 45 |
| Basal CCK, pmol/L | 0.314 | **0.038** | 44 | 0.227 | 0.138 | 44 | 0.091 | 0.555 | 44 | 0.272 | 0.075 | 44 |
| CCK iAUC, pmol/L*min | 0.193 | 0.203 | 45 | 0.095 | 0.535 | 45 | 0.122 | 0.424 | 45 | 0.171 | 0.262 | 45 |
| Data presented as means ± SD. SWL: suboptimal weight loss. OWL: optimal weight loss. AG: acylated ghrelin. CCK: cholecystokinin. GLP-1: total glucagon-like peptide-1 PYY: total peptide YY. DTE: desire to eat. PFC: Prospective food consumption. PFS-FA: food available. PFS-FP: food present. PFS-FT: food tasted. PFA-AS: aggregated domain. iAUC: incremental area under the curve. | | | | | | | | | | | | |
