# Supplementary material for: Homeostatic Appetite and Hedonic Hunger 13 Years After Roux-en-Y Gastric Bypass: Potential Associations and Predictive Value in Determining Long-Term Weight Loss Outcomes
Source: Obes Surg. 2025 Jun 9;35(7):2719–26. doi: 10.1007/s11695-025-07955-w (PMC12270954; doi:10.1007/s11695-025-07955-w)
Supplement: Supplementary file 2 — (DOCX 33.0 KB) [file 11695_2025_7955_MOESM2_ESM.docx]

|  | | | | | | | | | | | | | | | | | | | |
| --- | --- | --- | --- | --- | --- | --- | --- | --- | --- | --- | --- | --- | --- | --- | --- | --- | --- | --- | --- |
| Supplementary table 2. Correlations between subjective appetite ratings and hedonic hunger after RYGB | | | | | | | | | | | | | | | | | | | |
|  | PFS – FA | | | | PFS-FP | | | | | PFS-FT | | | | | PFS-AS | | | | |
|  | r | P | n | | r | | P | | n | r | | P | n | | r | | P | | n |
| Fasting hunger, mm | 0.228 | 0.136 | | 44 | 0.208 | 0.176 | | 44 | | 0.093 | 0.549 | | | 44 | 0.226 | 0.140 | | 44 | |
| Hunger iAUC, mm*min | -0.041 | 0.791 | | 45 | -0.043 | 0.779 | | 45 | | 0.056 | 0.713 | | | 45 | -0.018 | 0.907 | | 45 | |
| Fasting fullness, mm | -0.156 | 0.311 | | 44 | -0.009 | 0.952 | | 44 | | -0.079 | 0.610 | | | 44 | -0.100 | 0.519 | | 44 | |
| Fullness iAUC, mm*min | 0.070 | 0.647 | | 45 | 0.033 | 0.829 | | 45 | | -0.170 | 0.265 | | | 45 | -0.014 | 0.929 | | 45 | |
| Fasting DTE, mm | 0.350 | **0.020** | | 44 | 0.110 | 0.479 | | 44 | | 0.179 | 0.246 | | | 44 | 0.264 | 0.084 | | 44 | |
| DTE iAUC, mm*min | -0.126 | 0.410 | | 45 | 0.040 | 0.792 | | 45 | | -0.110 | 0.471 | | | 45 | -0.075 | 0.623 | | 45 | |
| Fasting PFC, mm | 0.505 | **<0.001** | | 44 | 0.295 | 0.052 | | 44 | | 0.141 | 0.362 | | | 44 | 0.403 | **0.007** | | 44 | |
| PFC iAUC, mm*min | -0.092 | 0.547 | | 45 | -0.005 | 0.974 | | 45 | | -0.063 | 0.681 | | | 45 | -0.065 | 0.672 | | 45 | |
| Data presented as means ± SD. DTE: desire to eat. PFC: Prospective food consumption. PFS-FA: food available. PFS-FP: food present. PFS-FT: food tasted. PFA-AS: aggregated domain. iAUC: incremental area under the curve. | | | | | | | | | | | | | | | | | | | |
