# Supplementary material for: Homeostatic Appetite and Hedonic Hunger 13 Years After Roux-en-Y Gastric Bypass: Potential Associations and Predictive Value in Determining Long-Term Weight Loss Outcomes
Source: Obes Surg. 2025 Jun 9;35(7):2719–26. doi: 10.1007/s11695-025-07955-w (PMC12270954; doi:10.1007/s11695-025-07955-w)
Supplement: Supplementary file 3 — (DOCX 22.1 KB) [file 11695_2025_7955_MOESM3_ESM.docx]

| Supplementary table 3. Multivariate linear regression models predicting TWL after RYGB | | | |
| --- | --- | --- | --- |
| Models | ß-coefficient (95% CI) | P value | Adjusted R^2^ |
| A. |  | 0.221 | 0.051 |
| Constant | 19.030 (-42.623, 80.68 4) | 0.536 |  |
| Age | -0.548(-0.253,1.349) | 0.174 |  |
| Sex | -10.266 (-27.827, 7.294) | 0.244 |  |
| Preoperative BMI | -0.452 (-1.444, 0.540) | 0.362 |  |
| PFS-FP | 2.8E^-2^ (-6.074, 6.130) | 0.993 |  |
| GLP-1 tAUC | 1.0E^-3^ ( -0.002, 0.004) | 0.645 |  |
|  |  |  |  |
| B. |  | 0.132 | 0.085 |
| Constant | 35.008 ( -30.298, 100.315) | 0.285 |  |
| Age | 0.594 ( -0.177, 1.365) | 0.127 |  |
| Sex | -8.920( -26.180, 8.341) | 0.302 |  |
| Preoperative BMI | -0.560( -1.551, 0.431) | 0.260 |  |
| PFS-AS | -4.855 ( -13.026, 3.316) | 0.237 |  |
| GLP-1 tAUC | 1.12E^-4^ ( -0.003, 0.003) | 0.938 |  |
|  |  |  |  |
| C. |  | 0.137 | 0.083 |
| Constant | 21.055 ( -39.617, 81.728) | 0.487 |  |
| Age | 0.447 ( -0.270, 1.164) | 0.215 |  |
| Sex | -11.113 ( -28.401, 6.175) | 0.201 |  |
| Preoperative BMI | -0.451 ( -1.423, 0.521) | 0.354 |  |
| PFS-FP | 0.684 ( -5.178, 6.546) | 0.815 |  |
| GLP-1 iAUC | 0.002 ( -0.001, 0.004) | 0.217 |  |
|  |  |  |  |
| D. |  | 0.098 | 0.103 |
| Constant | 35.073 (-29.578, 99.724) | 0.279 |  |
| Age | 0.472 (-0.230, 1.174) | 0.182 |  |
| Sex | -9.766 (-26.887, 7.355) | 0.256 |  |
| Preoperative BMI | -0.526 (-1.500, 0.448) | 0.282 |  |
| PFS-AS | -3.851 (-11.880, 4.177) | 0.338 |  |
| GLP-1 iAUC | 0.001 (-0.001, 0.004) | 0.377 |  |
|  |  |  |  |
| E. |  | 0.166 | 0.070 |
| Constant | 27.778 (-36.120, 91.676) | 0.385 |  |
| Age | 0.651 (0.016, 1.287) | 0.045 |  |
| Sex | -13.105 (-31.585, 5.376) | 0.159 |  |
| Preoperative BMI | -0.569 (-1.566, 0.428) | 0.255 |  |
| PFS-FP | 0.227 (-5.539, 5.993) | 0.937 |  |
| Hunger tAUC | -0.001 (-0.003, 0.001) | 0.318 |  |
|  |  |  |  |
| F. |  | 0.116 | 0.093 |
| Constant | 38.777 (-27.429, 104.983) | 0.243 |  |
| Age | 0.614 (-0.017, 1.246) | 0.056 |  |
| Sex | -10.928 (-29.381, 7.526) | 0.238 |  |
| Preoperative BMI | -0.606 (-1.591, 0.379) | 0.220 |  |
| PFS-AS | -4.068 (-12.298, 4.162) | 0.324 |  |
| Hunger tAUC | -0.001(-0.003, 0.001) | 0.550 |  |
| TWL: Total weight loss. tAUC: total areal under the curve. GLP-1: total glucagon-like peptide-1. DTE: desire to eat. PFC: Prospective food consumption. PFS-FA: food available. PFS-FP: food present. PFS-FT: food tasted. PFA-AS: aggregated domain. Variance inflation factors (VIF) <1.8. | | | |
